# SegVir: Reconstruction of Complete Segmented RNA Viral Genomes from Metatranscriptomes

**DOI:** 10.1093/molbev/msae171

**Published:** 2024-08-13

**Authors:** Xubo Tang, Jiayu Shang, Guowei Chen, Kei Hang Katie Chan, Mang Shi, Yanni Sun

**Affiliations:** Department of Electrical Engineering, City University of Hong Kong, Kowloon, Hong Kong (SAR), China; Department of Information Engineering, The Chinese University of Hong Kong, New Territories, Hong Kong (SAR), China; Department of Electrical Engineering, City University of Hong Kong, Kowloon, Hong Kong (SAR), China; Department of Electrical Engineering, City University of Hong Kong, Kowloon, Hong Kong (SAR), China; Department of Biomedical Sciences, City University of Hong Kong, Kowloon, Hong Kong (SAR), China; State Key Laboratory for Biocontrol, School of Medicine, Shenzhen Campus of Sun Yat-sen University, Sun Yat-sen University, Shenzhen 518107, China; Department of Electrical Engineering, City University of Hong Kong, Kowloon, Hong Kong (SAR), China

**Keywords:** segmented RNA viruses, genome reconstruction, metatranscriptomes

## Abstract

Segmented RNA viruses are a complex group of RNA viruses with multisegment genomes. Reconstructing complete segmented viruses is crucial for advancing our understanding of viral diversity, evolution, and public health impact. Using metatranscriptomic data to identify known and novel segmented viruses has sped up the survey of segmented viruses in various ecosystems. However, the high genetic diversity and the difficulty in binning complete segmented genomes present significant challenges in segmented virus reconstruction. Current virus detection tools are primarily used to identify nonsegmented viral genomes. This study presents SegVir, a novel tool designed to identify segmented RNA viruses and reconstruct their complete genomes from complex metatranscriptomes. SegVir leverages both close and remote homology searches to accurately detect conserved and divergent viral segments. Additionally, we introduce a new method that can evaluate the genome completeness and conservation based on gene content. Our evaluations on simulated datasets demonstrate SegVir’s superior sensitivity and precision compared to existing tools. Moreover, in experiments using real data, we identified some virus segments missing in the NCBI database, underscoring SegVir’s potential to enhance viral metagenome analysis. The source code and supporting data of SegVir are available via https://github.com/HubertTang/SegVir.

## Introduction

RNA viruses are a class of viruses whose genetic material is RNA. They can infect a wide range of organisms, from bacteria to humans (Vincent and Vardi 2023). Some of the most notorious pathogens are RNA viruses, such as SARS-CoV-2 for COVID-19 and HIV for AIDS (Cevik et al. 2020; Zhang et al. 2020). RNA viruses exhibit high diversity and can be classified into distinct groups based on various criteria, including genome structure, replication strategy, and the associated diseases. In this work, our focus is segmented RNA viruses, characterized by genomes that are fragmented into multiple segments (Dadonaite et al. 2019). The number of segments of known viruses can vary from 2 to 16. For example, influenza A virus and hantavirus are segmented RNA viruses containing eight and three segments, respectively (Bouvier and Palese 2008; Vaheri et al. 2013). However, our understanding of the prevalence of segmentation in RNA viruses is incomplete and many segmented viruses may remain undiscovered. This limitation highlights the need for improved methods to detect and characterize the full diversity of segmented RNA viruses.

The identification of segmented RNA viruses holds significant importance in virology and public health, as these viruses exhibit high variability and adaptability (Villa et al. 2021). They can generate new combinations of genomes through reassortment, resulting in the emergence of new subtypes or strains that can lead to epidemics or pandemics (Vijaykrishna et al. 2015; McDonald et al. 2016). Identifying segmented RNA viruses also contributes to understanding their origins, evolution, transmission, and pathogenesis, providing a basis for the development of vaccines and drugs. Based on our literature review of published RNA viruses, most known segmented RNA viruses belong to 40 families. Meanwhile, multiple recent studies indicate the likely existence of undiscovered segmented RNA viruses. Gilbert et al. (2019) and Cook et al. (2013). In particular, there are reports of new segmented RNA viruses in insects (Käfer et al. 2019).

Currently, metagenomic sequencing provides a comprehensive way to sequence viruses in a sample (Zhang et al. 2021a). With additional steps for RNA extraction and cDNA synthesis, metatranscriptomic sequencing (abbreviated as metagenomic sequencing hereafter) allows for sequencing all RNA molecules present in a sample, enabling the rapid identification of novel RNA viruses (Ladner 2021). However, several challenges hinder the reconstruction of complete genomes of segmented RNA viruses from the metagenomes (Smits et al. 2014). The first challenge stems from the high sequence diversity of some segments, which complicates the process of obtaining all segments through alignment-based methods. For instance, RNA-dependent RNA polymerase (RdRp), recognized as the most conserved protein among RNA viruses, is often employed as a marker for RNA virus identification (Ahlquist 2002; Charon et al. 2022). As a result, segments containing RdRp are usually easier to identify. In contrast, other segments, such as those encoding for viral coat proteins, tend to exhibit lower conservation and thus pose a greater challenge for their identification.

In addition to that, segmented viruses cannot be assembled into a complete contig by metagenomic assembly tools. Although reconstructing segmented viral genomes shares some resemblance with the contig binning problem, binning tools often fail to reconstruct the segmented viruses because k-mer distributions of different segments can vary significantly. Finally, the complex composition of metagenomes increases the risk of mistaking nonviral contigs from hosts or bacteria as viral contigs because host genomes can integrate certain genes from infected RNA viruses (Zhang et al. 2021b). For instance, the nonretroviral endogenous viral elements (nrEVEs) are the nonretroviral integrated virus elements in genomes and have been found in certain mosquitoes (Palatini et al. 2022; Veglia et al. 2023). Despite quality control steps, not all reads from the host can be filtered out, so some assembled contigs will contain nrEVE and thus affect downstream analysis. While some tools, such as CheckV (Nayfach et al. 2021), have been developed to identify host contamination, their efficacy is limited by several factors. These include high mutation rates, the presence of multiple segments, and the potential for recombination events, all of which restrict CheckV’s ability to detect all nrEVEs in samples. Empirical evidence suggests that the number of nrEVEs is currently underestimated for arthropods (Huang et al. 2023), indicating that CheckV and similar tools cannot comprehensively capture the extent of nrEVE integration into host genomes. Another source of misclassification is bacteria that are infected by segmented RNA phages.

Multiple tools have been developed for identifying RNA viruses, including VirSorter2 (Guo et al. 2021), VirFinder (Ren et al. 2017), VirBot (Chen et al. 2023), and viralVerify (Antipov et al. 2020). However, none of these tools can reconstruct complete or near complete segmented RNA viruses. These tools primarily focus on virus identification without providing more detailed information on the segments, especially for learning-based tools. Because we know which taxonomic groups contain segmented viruses, taxonomy classification tools can be applied for reconstructing segmented viruses, such as CAT (Von Meijenfeldt et al. 2019), MMseqs (Steinegger and Söding 2017), Kaiju (Menzel et al. 2016), and Kraken2 (Wood et al. 2019). After obtaining the taxonomy of each contig, the segmented RNA viruses can be reconstructed based on the obtained taxonomy information. CAT aligns the query to a protein database and utilizes the Lowest Common Ancestor (LCA) algorithm to estimate the taxon. MMseqs and Kraken2 employ k-mer-based methods, while Kaiju uses the Maximum Exact Match (MEM) approach to assign taxonomy. These tools’ performance on reconstructing segmented viruses has not been thoroughly evaluated. We will provide a comprehensive evaluation in this work.

Evaluating the completeness of the identified viral genomes is equally essential. Tools such as viralComplete (Antipov et al. 2020), VIBRANT (Kieft et al. 2020), and CheckV have been developed to assess the completeness of metagenome-assembled viral genomes. VIBRANT uses metrics such as circularity, nucleotide replication, and viral hallmark proteins to estimate the quality of predicted viral sequences, representing their completeness. Both viralComplete and CheckV compute the “similarity” of a given contig to the reference viral genomes and estimate completeness by comparing it with the closest one or group of reference genomes. However, these tools are designed for nonsegmented genomes. Evaluating the completeness of segmented viruses must accommodate the variability of segment numbers. Even within the same viral family, the number of segments can vary. For instance, in the family *Spinareoviridae*, the number of segments ranges from 8 to 16. Therefore, the completeness of a virus cannot be determined solely based on the number of contigs found. Consequently, a new method needs to be developed to evaluate the completeness of segmented viral genomes.

Very limited tools are available for identifying segmented viruses from metagenomic data. One such tool uses iterative BLAST (Zhang et al. 2024). This method leverages an important feature of segmented viruses: the untranslated regions (UTRs), which are often conserved within the segments of many viruses. In this approach, a protein database is used to iteratively identify viral contigs through BLASTx (iBLASTx), while BLASTn is used iteratively (UTR-iBLASTn) to search for contigs that share similar UTRs. Through iterative searching, this method can detect distantly related sequences in the sample. However, it still cannot determine the completeness of the identified segmented viruses.

To provide a more accurate and complete catalog of segmented viruses from various metatranscriptomes, we developed a tool named SegVir for identifying segmented viruses and reconstructing their complete genomes. SegVir takes into account the conservation levels of different segments and employs corresponding alignment strategies to detect both well-conserved segments and diverged segments. To minimize false positives (FPs), SegVir incorporates references from possible contamination sources, such as nonsegmented viruses and hosts. Moreover, we measure the completeness of recovered genomes based on the gene content of identified viral segments from the same family. We evaluate SegVir under various scenarios. First, we compared the sensitivity of different tools in identifying segmented viruses that share low similarity with reference viruses. Second, we generated multiple simulated metagenomes to evaluate the sensitivity and precision of SegVir. Finally, we deployed SegVir to analyze real data, evaluating its capacity to recover complete genomes of segmented viruses and identify novel viruses. The results showed that SegVir outperformed existing tools in terms of both sensitivity and precision. Additionally, SegVir successfully recruited missing segments and accurately identified previously unknown viruses, highlighting its potential for comprehensive segmented viral genome analysis in metagenomes.

## Materials and Methods

**Fig. 1.**
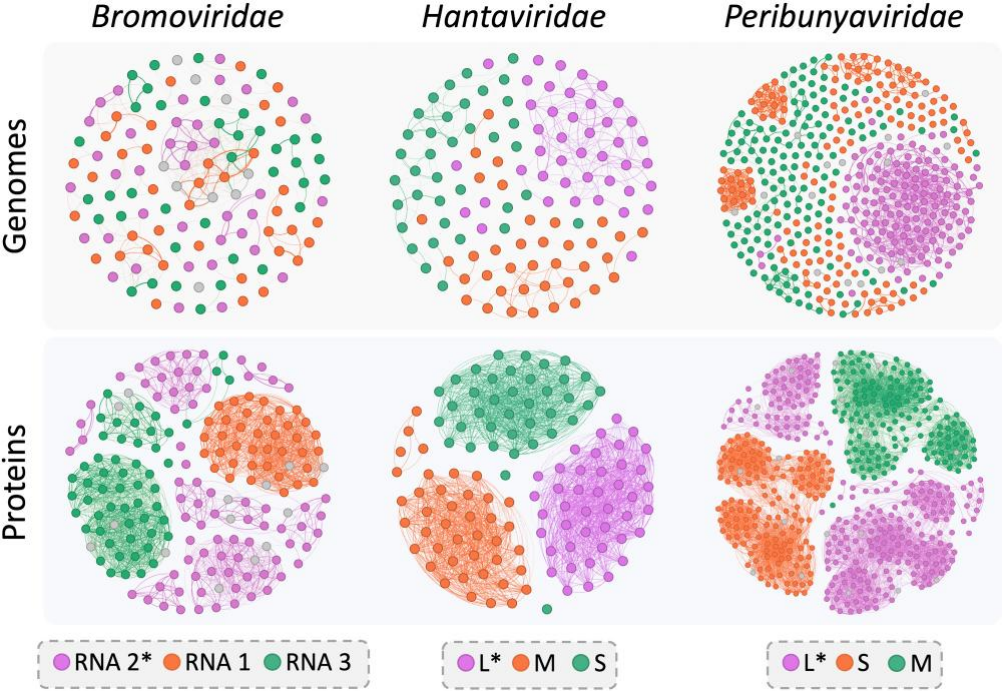
The similarity graphs of *Bromoviridae*, *Hantaviridae*, and *Peribunyaviridae* at both the genome (top) and protein levels (bottom). Gephi (Bastian et al. 2009) was employed to visualize the graph, along with the Fruchterman–Reingold (Fruchterman and Reingold 1991) algorithm to arrange nodes. Different colors represent distinct segments, with the IDs of the segments displayed in the legend. Gray nodes indicate that their segment information is unknown. Segments marked with asterisks in the legend denote those encoding RdRps.

Homology search is still the major methodology for segmented RNA virus identification. Thus, we first examine the conservation level of viral genomes and viral proteins for segmented viruses. This analysis can help us select the appropriate databases and design the key components in the pipeline. We first download the completed genomes and their encoded proteins for each viral family from RefSeq. For a viral family that is enriched with segmented viruses, we analyze the sequence similarity by constructing similarity graphs for genomes and proteins within each family, respectively. In a similarity graph, nodes represent sequences (segments or proteins), and edges denote sequence similarity, which is the product of pairwise alignment identity and coverage by BLAST/BLASTP. As an example, the similarity graphs for viruses in three tri-segmented families, including *Hantaviridae*, *Bromoviridae*, and *Peribunyaviridae*, are shown in Fig. 1. Based on these similarity graphs, we made the following observations.

1. Proteins exhibit better conservation than segments in nucleotide, as shown by the edge density and clear clustering of nodes in the “protein similarity graph”, whereas many of the nodes in the “genome similarity graph” do not share an edge due to low similarity. For instance, within *Bromoviridae*, only a few segments labeled as “RNA 3” can be aligned by BLAST. Thus, our algorithm will use protein-based homology search for identifying segmented viruses.
2. Different segments display varying levels of conservation. For example, segment L in both *Hantaviridae* and *Peribunyaviridae* exhibits higher similarity than other segments. This phenomenon is more obvious at the protein level. For instance, in *Bromoviridae*, proteins on segment “RNA 1” are grouped in one cluster, while proteins on other segments are divided into several clusters. Similarly, segment M in *Hantaviridae* and segment L in *Peribunyaviridae* also display higher diversities than other segments. The reason is 2-fold. One is that some segments encode multiple proteins (e.g. *Bromoviridae* “RNA 3”). The other is that some proteins exhibit a high degree of diversity (e.g. *Peribunyaviridae* L). This suggests that pairwise alignment may not identify all segments, necessitating remote homology search.

### Pipeline of Reconstructing the Segmented RNA Viruses

Based on the above analysis, we design our tool SegVir for segmented RNA virus detection. It takes assembled contigs as input and outputs segmented viruses with completeness and conservation scores, which quantify the completeness of the identified virus and its conservation with respect to reference viruses, respectively.

**Fig. 2.**
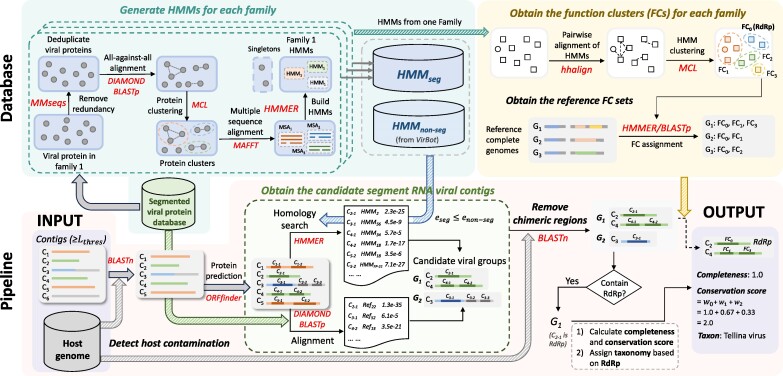
The pipeline of building the reference databases and reconstructing segmented RNA viruses. We applied different background colors to distinguish three main components. Upper left block: constructing protein family pHMM database. The cylindrical shapes denote three databases: viral protein database for segmented RNA viruses, ${\text{HMM}}_{\mathrm{seg}}$ for segmented viruses, and ${\text{HMM}}_{\mathrm{nonseg}}$ for other viruses. Upper right block: the establishment of the function cluster (FC) database, along with the construction of reference FC sets. Lower block: the pipeline of SegVir.

For each input contig, we use pairwise sequence alignment and profile hidden Markov model (pHMM)-based methods for close and remote protein homology search, respectively. The homology search results will be used to assign each contig to a viral family based on the taxonomic label of the best match. In our study, we found that the majority of the data contained only one segmented virus from one family. Thus, we group contigs bearing the same family label as the same segmented virus. In rare cases where multiple segmented viruses from the same family are present within the same sample, sequencing coverage will be used to distinguish them. Figure 2 illustrates the pipeline, and we elaborate on the main steps below.

1. Filter out the input contigs shorter than $L_{\mathrm{thres}}$. For all experiments in this paper, we set $L_{\mathrm{thres}}$ to 300 nucleotides (nt). Users can adjust $L_{\mathrm{thres}}$ conveniently in the tool. Short contigs often lack sufficient information for accurate identification. Moreover, segments in segmented RNA viruses are typically longer than 1,000 nt. Even if the complete segment cannot be assembled, a threshold of 300 nt in length can still be employed to retrieve as many contigs as possible.
2. Remove host-borne contigs by aligning these with the host genomes using BLASTn (Camacho et al. 2009). Contigs that exhibit over 95% identity and 95% coverage are removed. We applied this stringent cutoff to avoid removing true viral contigs.
3. Employ ORFfinder to predict proteins from the filtered contigs, and align predicted proteins against the reference databases using both DIAMOND BLASTP (Buchfink et al. 2015) and HMMER (Finn et al. 2011). Once the protein aligns to the reference database by either tool, the corresponding contig is considered as a candidate segmented viral contig. As the pHMMs from ${\text{HMM}}_{\mathrm{seg}}$ are built for each family, the aligned contigs with either BLASTP or HMMER can be assigned with the corresponding family label. Meanwhile, ${\text{HMM}}_{\mathrm{nonseg}}$ will be utilized to remove nonsegmented viral contigs. Then, viral contigs from the same family will be grouped together as input to the next step.
4. As the removal of host-borne contigs step (Step 2) employed a very stringent criterion, we performed an addition step to detect and eliminate host contamination from chimeric contigs. Based on the alignment results, if a contig exhibits alignment to both the host and viral databases, with distinct regions aligned to each, it is considered a chimeric contig. Specifically, if a region of the contig aligns to host genomes with an identity >80% and an *e*-value smaller than 1e−5, we consider that this region is from the host. In such cases, only the segment of the contig that aligns with the viral database will be retained, while the segment aligned to the host genome will be discarded.
5. Verification and completeness estimation. For the candidate viral groups that have been checked for host contamination, we examine whether each group contains contigs encoding RdRp, with an aligned *e*-value below $10^{-5}$ and a length >400 nt. Only groups containing RdRp are considered to have contigs originating from RNA viruses because RdRp serves as a reliable marker for RNA virus identification. Subsequently, after obtaining the verified viral group, we calculate the completeness of the recovered viral genomes and the conservation score. Furthermore, to enhance the result interpretation, we perform a species-level taxonomic assignment for the contigs within the group based on RdRps, and the detailed method can be found in supplementary S1.1, Supplementary Material online.

Below, we elaborate on two key components in the pipeline (Fig. 2): (i) building a reference database to identify candidate viral contigs and (ii) evaluating the completeness of the recovered genomes.

### Building Reference Databases for Identifying Segmented Viral Contigs

To leverage close and remote protein-based homology search for segmented viral contig identification, we need to construct the reference databases. Specifically, we constructed reference sequence and pHMM databases (named ${\text{HMM}}_{\mathrm{seg}}$) for pairwise and pHMM-based homology search, respectively. Furthermore, to mitigate the risk of misclassifying nonsegmented viruses as segmented viruses, we constructed an additional pHMM (Finn et al. 2011) database for nonsegmented viruses, referred to as ${\text{HMM}}_{\mathrm{nonseg}}$.

We first constructed a DIAMOND BLASTP database for pairwise alignment using all the reference segmented RNA viral proteins. Compared with BLAST (Altschul et al. 1997), DIAMOND offers significantly improved computational speed, albeit with a minor trade-off in sensitivity. Meanwhile, we utilized these proteins to build the ${\text{HMM}}_{\mathrm{seg}}$ database for each virus family so that we could use the pHMMs for family-level classification. To construct the ${\text{HMM}}_{\mathrm{seg}}$ database, we employed a graph clustering method to cluster proteins in each family. First, we used MMseqs to remove redundant proteins that had a similarity and coverage of 90% or more. Then, we performed an all-against-all alignment for the nonredundant proteins to build the graph, where nodes represent proteins and an edge is created between two proteins if their alignment has an *e*-value below 10e−5. Subsequently, we applied the Markov Cluster Algorithm (MCL) (Van Dongen 2008) to cluster the proteins, retaining only clusters containing more than two proteins. Proteins that are not clustered are treated as singletons. We then used MAFFT (Katoh and Standley 2013) to generate the multiple sequence alignment (MSA) for each cluster and constructed pHMM. Figure 2 shows the databases and the pipeline of building ${\text{HMM}}_{\mathrm{seg}}$.

We utilized pHMM profiles from the VirBot (Chen et al. 2023) and excluded all models with training sequences from segmented viruses. A total of 724 HMM profiles were collected for ${\text{HMM}}_{\mathrm{nonseg}}$. If a contig can be aligned to both ${\text{HMM}}_{\mathrm{seg}}$ and ${\text{HMM}}_{\mathrm{nonseg}}$, SegVir examines the bit-score. Only a contig with smaller bit-scores with ${\text{HMM}}_{\mathrm{seg}}$ than with ${\text{HMM}}_{\mathrm{nonseg}}$ is classified as a nonsegmented viral contig.

### Estimating the Completeness and Conservation of Recovered Genomes

There is no standard definition for the completeness evaluation of segmented viruses. Using the number of segments is not ideal because of the variation of segment numbers for viruses in the same family. For instance, *Spinareoviridae* viruses can have as few as 8 or as many as 16 segments. In addition, some important proteins, such as RdRp, can be present in different segments. Thus, using segments cannot distinguish the case of missing a well-conserved or rare protein. On the other hand, the number of proteins associated with important functions in the segmented viruses is usually fixed. For example, segmented viruses within the *Arenaviridae* family exhibit varying segments (2 or 3), yet all encoded proteins are associated with three classes of functions: infection, genome packaging, and genome replication. Therefore, we propose a completeness and conservation estimation based on protein functions. Specifically, we construct “function clusters” (FCs) where each cluster contains proteins of similar functions. For an identified virus, we assess its completeness by examining whether it contains all necessary FCs. Meanwhile, we can estimate its conservation relative to the reference database based on the frequency of FC occurrence in the reference genomes.

As shown in Fig. 1, proteins of the same function can be divided into multiple protein clusters (PCs). To construct FCs, we need to further group PCs of the same function into one cluster. The PCs are previously derived using clustering on protein sequences. The most important component of grouping PCs into FCs is to establish an appropriate evaluation of the similarity between PCs. We thus evaluated three types of methods: sequence-based similarity, HMM-based similarity, and three-dimensional structural similarity, as shown in supplementary Table S1, Supplementary Material online. Detailed methods and results are presented in supplementary S1.2, Supplementary Material online. Based on the experiment, using hhalign (Steinegger et al. 2019) to compute HMM similarity followed by MCL clustering yielded FCs that are mostly consistent with the key protein functions in a viral family. Thus, we construct the FCs using the PC clustering results based on the pHMM–pHMM alignment score by hhalign.

In our constructed FCs, each protein or PC can be mapped to a unique FC. It is important to note that we performed joint clustering of PCs and singletons, resulting in FCs that may exclusively contain PCs, or include both. It is clear that the number of FCs is significantly less than the PCs, as FCs are constructed using remote homology search between pHMMs. These FCs are better representations of the key functions of proteins inside each viral family, providing a better baseline to evaluate the completeness of a new virus. Furthermore, for each FC, we extracted keywords from the protein descriptions as the annotations for the FCs. The numbers of PCs and FCs for all the families containing segmented RNA viruses from the NCBI virus database (release date: March 2024) are shown in supplementary Table S3, Supplementary Material online.

Upon obtaining the FCs, we annotated the FCs in each genome in the reference database using DIAMOND/HMMER, allowing to encode a genome into a set of FCs. We thus can construct a database containing multiple sets of FCs, where each set represents a complete genome. This database will be utilized to evaluate the completeness and conservation of a newly identified viral genome. For a query genome, after annotating its FCs, we first search for its best match in the reference FC database using set-based similarities. Intuitively speaking, the best match is a virus under two considerations: (i) high Jaccard similarity in terms of FCs and (ii) the best match should contain most if not all FCs in the query. The detailed calculation method is detailed in supplementary S1.3, Supplementary Material online. Once we identify the best match, we use the reference FC set to compute the completeness of the query virus. The ratio of the number of FCs in the query genome to the number in the best match is taken as the query genome’s completeness. As this completeness is computed based on protein functions, we named it as *FunC*.

In addition to assessing genome completeness, we introduce a scoring system to evaluate the relative conservation of query genomes compared to the reference database. We refer to this score as the conservation score (*ConS*). To compute *ConS*, we initially calculate the frequency of each FC across reference genomes, using it as a weight. Higher FC occurrence corresponds to greater weight, signifying higher conservation within genomes. We have plotted the distribution of FC weights and observed that it is not uniform. The weights are predominantly concentrated near the extremes of 0 and 1, as illustrated in supplementary Fig. S2, Supplementary Material online. For a given genome, we sum the weights of its included FCs to determine its *ConS*. We can compare the *ConS* of query genomes with that of reference genomes to assess the conservation of the query genome relative to the reference genome. Detailed methods for calculating the *ConS* are provided in supplementary S1.4, Supplementary Material online. Finally, for query genomes, we compute both their completeness and *ConS*, where completeness closer to 1 indicates better recovery, while a higher *ConS* suggests that this new virus contains well-conserved proteins.

## Results

We evaluated the performance of SegVir on simulated and real data. The descriptions of datasets in all experiments are shown in Table 1. We benchmarked SegVir with iBLASTx (Zhang et al. 2024) and other tools that can be customized for this purpose. We compared the sensitivity of different tools in identifying different segments. Next, we compared these tools’ sensitivity and precision on a set of simulated metatranscriptomes that contained multiple segmented RNA viruses and negative samples (hosts, bacteria, and nonsegmented RNA viruses). In the above two experiments, the reference sequences and the correspondingly derived pHMMs and FCs have no overlaps with the test sequences. We further tested SegVir’s ability to identify the missing segments of segmented RNA viruses in the public database. In the end, we evaluated SegVir using metatranscriptomes collected from mosquitoes. As for the above two experiments involving real data, we utilized the proteins from 40 families containing segmented RNA viruses (NCBI virus database March 2024) to build SegVir’s reference protein and pHMM database (supplementary Table S3, Supplementary Material online).

**Table 1:** The descriptions of datasets

Dataset	Description
Low-similarity dataset	To mimic the case of detecting diverged/novel segmented viruses, we partition the segmented viruses from *Orthomyxoviridae* into training and test sets so that the overall similarity between the two sets is minimized.
Simulated metatranscriptomes	Simulated mosquito metatranscriptomes containing segmented RNA viruses, the host, bacteria, and nonsegmented RNA viruses. The details of the reference can be found in supplementary Table S5, Supplementary Material online. To simulate data with increasing complexity, we generated three types of datasets labeled as I, II, and III, each comprising different compositions of organisms. For each type of dataset, we randomly generated five sets to ensure the reliability of the evaluation.
Missing segment datasets	We downloaded two SRA datasets from experiments that detect segmented RNA viruses. They are SRR16905247, collected by Mang Shi (Feng et al. 2022) from *Culex pipiens*, and SRR19790906, collected by Van Brussel et al. (2022) from *Pteropus poliocephalus*. Some of the segmented viruses contained only the segments encoding RdRp in the NCBI database. We assembled the contigs from the downloaded SRA data and utilized SegVir to identify the missing segments.
Real metatranscriptomes	The real mosquito metatranscriptomes, collected by Mang Shi et al., comprise samples from various locations and dates. We grouped the samples according to their dates and locations and performed coassembly to generate the contigs. We utilized SegVir to reconstruct the segmented RNA viruses from these samples. The accession numbers of SRA samples can be found in supplementary Table S7, Supplementary Material online.

### Performance on Identifying Different Segments in *Orthomyxoviridae*

One challenging case for reconstructing segmented viruses is to identify those diverged ones. In this experiment, we mimic this case by partitioning the sequences into reference and test sets with low similarities. Then, we evaluate how many of the segments can be identified by different tools, which can be quantified by sensitivity (or recall). As we do not have “negative” samples in this experiment, we do not measure precision here. We chose the largest family, *Orthomyxoviridae*, in this experiment because of the number of available sequences and also the number of segments. This family contains up to eight segments, and the influenza A and B viruses are both within this family. To construct this dataset, we downloaded the complete genomes of *Orthomyxoviridae* from RefSeq.

We first eliminated redundancy within the genome by concatenating the segments of the genome and using cd-hit-est to remove genomes with 99% identity and 99% coverage. Subsequently, we used dashing (Baker and Langmead 2019) to calculate the similarity between genomes. Dashing estimates the evolutionary distance between genomes by extracting k-mers from the genes and calculating the Jaccard index of the k-mer contents. We constructed a genome similarity graph in which each node represents a genome, and the edges are weighted based on the similarity between genomes. Once the graph was constructed, we divide the nodes into two sets so that the cross edges between the two sets are minimized. By doing so, we can minimize the similarity between the two sets. Then, the larger set was assigned as the reference data, while the smaller set was utilized as the test set. Finally, we obtained 342 training genomes and 51 test genomes. The training and test genomes are mainly composed of *Influenza B viruses* and *Influenza C viruses*, respectively. The details of the dataset are shown in supplementary Table S4, Supplementary Material online. When constructing the SegVir reference database, we obtained 17 PCs and 14 FCs.

**Fig. 3.**
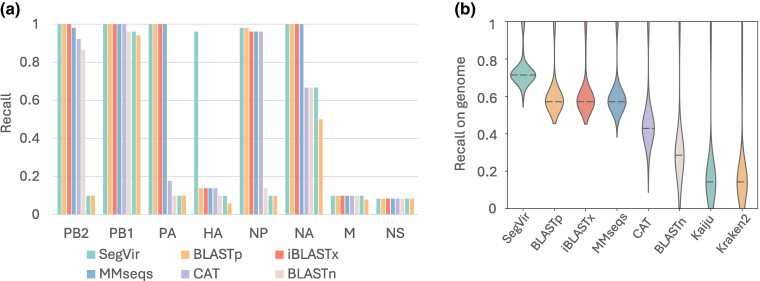
The performance of different tools for identifying different segments in *Orthomyxoviridae*. a) The recall of different tools on segments with different proteins. *X* axis: types of proteins. *Y* axis: ratio of identified contigs to the total number of sequences for a specified type of protein. PB2, polymerase basic 2; PB1, polymerase basic 1; PA, polymerase acidic; HA, hemagglutinin; NP, nucleoprotein; NA, neuraminidase; M, matrix; NS, nonstructural. b) The recall of all segments within genomes of different tools, which is the ratio of identified segments to the total number of segments of a viral genome. *X* axis: different tools.

Here, we compared the performance of SegVir with seven other tools: DIAMOND BLASTp, iBLASTx, MMseqs, CAT, Kaiju, Kraken2, and BLASTn. Apart from BLASTn, we utilized all the proteins encoded in the training genomes to construct the corresponding database. For BLASTn, we directly utilized the viral sequences as the reference. We first grouped segments based on their main coding protein and then evaluated the recall of different tools on the segments encoding different proteins, as shown in Fig. 3a. There is a significant variation in the sensitivity of different tools, and the ratio of segments with different proteins being identified also varies greatly. Comparing the results of SegVir and BLASTp, we can see that pHMM-based alignment significantly contributes to the high sensitivity, as HMMER detected 43 more hemagglutinin (HA) segments than BLASTp. SegVir also significantly outperforms iBLASTx and MMseqs on HA segments, with sensitivities of 96.0% versus 13.7% and 13.7%, respectively. This is particularly important as the HA protein is crucial for guiding influenza vaccine development. Both SegVir iBLASTx, and MMseqs show high sensitivity in identifying sequences of PB2, PB1, and PA segments, which constitute RdRp. The sensitivity of all tools for matrix (M) and nonstructural (NS) segments is not high. This is due to that training and test data are dominated by different classes of viruses, and the similarity of these proteins between the training and test data is relatively low. Segments 6 and 7 typically encode neuraminidase and matrix protein, respectively. These two proteins may exhibit significant differences across different viruses, leading to low sensitivities. For instance, among various influenza A viruses, there exist nine distinct neuraminidase subtypes (N1–N9), along with N10 and N11 subtypes found in bats (McAuley et al. 2019).

While Fig. 3a shows the recall of segments with different functions, Fig. 3b summarizes the recall of all segments within genomes (ratio of identified segments to the total number of segments) of different tools. SegVir recovered the most complete virus, followed by MMseqs, CAT, BLASTn, Kaiju, and Kraken. We also estimated the completeness of the viral genomes identified by different tools using our FC-based method, as shown in supplementary Fig. S3, Supplementary Material online. We can see that the ranking of the completeness score of different tools is in line with their recall of all segments, proving that our gene content-based method can estimate the viral genomes’ completeness.

####  

##### Leave-one-family-out experiment

In addition to studying the sensitivity of identifying viruses within the same family, we also designed a leave-one-out test by family to evaluate SegVir’s ability to identify novel viruses. In this experiment, we used one family as the test set and all other families as the reference dataset. Our results showed that most new viruses could be identified and aligned with other families within the same order. We have included this experiment in supplementary S1.5, Supplementary Material online.

### Experiments on the Simulated Metatranscriptomes

Insects can host many segmented viruses. Among them, mosquitoes serve as important vectors for transmitting infectious diseases such as dengue fever and Zika virus. Therefore, studying viruses harbored by these insects holds significant implications for public health. Additionally, researchers have discovered a high abundance of nonretroviral integrated RNA viral elements (nrEVEs) in mosquitoes, which can interfere with virus detection. Hence, to comprehensively assess the performance of SegVir, we constructed a set of simulated metatranscriptomes from mosquitoes. The simulated data include host genomes, segmented and nonsegmented RNA viruses with mosquitoes as hosts, as well as bacteria infected by segmented RNA phages. To better simulate real-world scenarios, we referred to the real mosquito metagenomes in He et al. (2021). Based on the final analysis results from this work (He et al. 2021), we selected three host genomes (names are shown in supplementary Table S5, Supplementary Material online), eight segmented RNA viruses (derived from four families, with each family containing two viruses), and 15 nonsegmented RNA viruses for read simulation. Additionally, based on Hamze et al. (2023) and Charan et al. (2013), we selected six bacteria infected by segmented RNA phages from the *Pseudomonas* family. Furthermore, we employed the aforementioned graph-based data partition algorithm to ensure the lowest possible similarity between the reference and test segmented RNA viruses. The details of the reference database are shown in supplementary Table S5, Supplementary Material online.

To gain a better understanding of the interference originating from hosts and bacteria, we evaluated the similarity between the segmented RNA viruses and the host genome as well as with the bacterial genomes. Supplementary Figs. S4 and S5, Supplementary Material online depict the similarities and coverage derived from the pairwise alignments. Supplementary Fig. S4, Supplementary Material online shows that the alignment length between viruses and hosts is predominantly between 0 and 250 bp, with an identity exceeding 80%. Regarding the bacteria, the alignment lengths are primarily around 150 bp, with identities ranging from 25% to 40%.

To simulate data with varying complexity, we constructed three datasets with different compositions of organisms. The compositions are detailed in Table 2. Each dataset simulates data from a distinct host, and the number of bacteria and viruses increases from Dataset I to III. We utilized the InSilicoSeq (Gourlé et al. 2019) to simulate the metatranscriptomes with a coverage of 10×. For reliable evaluation, we generated five datasets for each setup.

**Table 2:** The composition of the different simulated mosquito datasets

Dataset	Host	# Bac	# Seg	# Nonseg
I	*Culex pipiens pallens*	2	1×1	5
II	A*edes aegypti*	4	2×2	10
III	*Anopheles stephensi*	6	2×4	15

# Bac, the number of bacteria; # Seg, the number of segmented RNA viruses, represented by *the number of sampled viruses* × *the number of sampled families*. For example, 2×4 represents eight segmented viruses, with two from each of four families. # Nonseg, the number of nonsegmented RNA viruses.

**Fig. 4.**
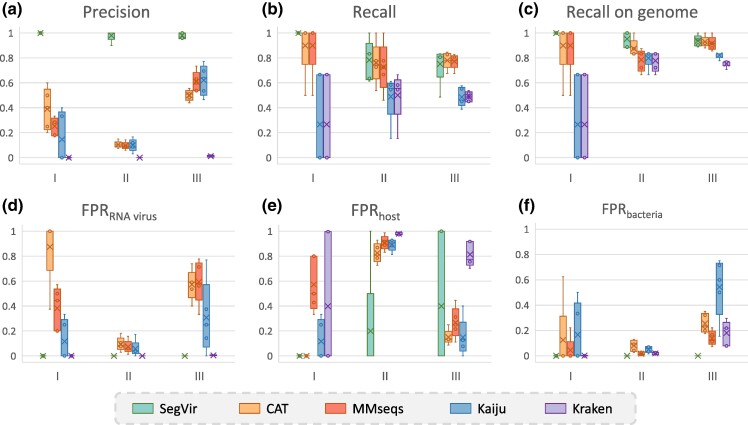
Performance of different tools on the simulated mosquito metatranscriptomes. The *X* axis of all subgraphs is the dataset ID. a) The precision of different tools, which is the ratio of correctly identified segmented RNA viral contigs to the total number of identified contigs. b) The recall of different tools, which is the ratio of identified segmented RNA viral contigs to the total number of segmented RNA viral contigs. c) The recall on genome, which is the ratio of identified segments to the total number of segments of a viral genome. d)–f) The FP rate of RNA virus, host, and bacteria.

We compared the performance of our tool with four others: CAT, MMseqs, Kaiju, and Kraken2. For a fair comparison, we utilized the same reference dataset of segmented RNA viruses for all the tools. Additionally, we included the proteins of the hosts in the references of the benchmarked tools. To evaluate performance comprehensively, in addition to assessing precision, recall, and recall of all segments, we also evaluated the impact of different negative samples on precision. For three types of FPs (nonsegmented RNA virus, host, and bacteria), we calculated the corresponding false positive rate (FPR) using ${\text{FPR}}_{n}={\text{FP}}_{n}/({\text{FP}}_{n}+{\text{TN}}_{n})$. Here, *n* can be RNA virus, host, or bacteria, and ${\text{FP}}_{n}$ and ${\text{TN}}_{n}$ represent the number of *n* in FPs and true negatives (TNs), respectively. The performance of the different tools on the simulated datasets is presented in Fig. 4.

As shown in Fig. 4b, as sample complexity increases (from 1 to 3), there is a trend of decreasing recall among the tools. This is due to the increased challenge of identifying all segmented RNA viruses as their number increases. While SegVir does not have the highest recall on Dataset III, it recovers the most complete viral genomes, having the highest recall of all segments. The average estimated completeness of SegVir on Datasets I, II, and III is 1.0, 0.945, and 0.932, respectively. Moreover, the performance of some tools shows significant fluctuations on Data I due to the limited number of samples. This effect is particularly notable in Kaiju and Kraken2.

Figure 4a shows that SegVir achieves the highest precision across all datasets. In addition, precision does not correlate with sample complexity, and overall precision for all tools is lower on Dataset II. By analyzing the sources of FPs, we can observe that the reason for the lower precision on Data II is host contamination, which contributes to the FPs across different tools. For Data I and Data II, the sources of FPs differ among various tools. For SegVir and CAT, they are primarily influenced by the host and nonsegmented RNA viruses, respectively. Kraken mainly encounters FPs originating from the host. However, as the abundance of bacteria increases, the FPs for Kraken include more contigs from bacteria. Moreover, MMseqs shows a reduced susceptibility to bacterial influence, while Kaiju does not show a clear pattern in terms of the source of FPs.

### Performance in Identifying the Missing Segments in the NCBI Datasets

To evaluate the performance in recovering the complete genomes of segmented RNA viruses from the real data, we employed SegVir to identify the missing segments of some segmented RNA viruses available at NCBI. We search for such viruses using the following procedure. First, we downloaded all the segments from the NCBI Virus database and checked whether the non-RdRp segments were absent. Then, we checked the availability of the corresponding raw sequencing data at NCBI SRA to ensure we could assemble the contigs. In the final analysis, we selected two SRA datasets: SRR16905247, collected from *Culex pipiens* by Mang Shi (Feng et al. 2022), and SRR19790906, collected from *Pteropus poliocephalus* faecal by Van Brussel et al. (2022). These datasets are utilized for studying the viromes associated with their respective hosts using metatranscriptomics data. We employed metaspades (Nurk et al. 2017) to assemble contigs and applied SegVir to identify the missing segments, as shown in Table 3.

**Table 3:** The identified missing segments from the targeted SRA data

SRA	Virus	Family	# Seg	*FunC*	*ConS*	*Ref ConS*
	*XiangYun bunya-arena-like virus 1*	*Leishbuviridae*	1	0.67	1.33	1.67
SRR16905247	*Zhee Mosquito virus*	*Peribunyaviridae*	2	1.0	1.02	2.89
	*Enontekio phenui-like virus 2*	*Phenuiviridae*	1	Nan	Nan	Nan
SRR19790906	*Bat faecal associated nodavirus 1*	*Nodaviridae*	1	1.0	1.68	1.20
	*Bat faecal associated qinvirus-like virus 1*	*Qinviridae*	1	1.0	1.89	1.89

Virus, the names of viruses determined based on the RdRp segments; # Seg, the number of identified non-RdRp segments; *FunC*, the completeness of the virus; *ConS*, the conservation score of the virus; *Ref ConS*, the reference conservation score; Nan, the score is unavailable.

In SRR16905247, we identified four previously unreported segments of three viruses that contain RdRp in the NCBI database. Among them, two missing segments were identified in *Zhee Mosquito virus* (ZMV) with corresponding estimated completeness scores of 1. However, the *ConS* of ZMV is lower than reference *ConS*. This implies that ZMV may be a relatively novel virus with regard to the reference database. Regarding *XiangYun bunya-arena-like virus 1* (XyBLV1), we identified one missing segment with an estimated completeness score of 0.67, suggesting that the viral genome identified is not yet complete. The virus belongs to the family *Leishbuviridae*, which typically comprises three segments. The missing segment of XyBLV1 was identified using HMMER, and considering the incomplete identification of all segments, this virus has lower *ConS* than the reference *ConS*. As for *Enontekio phenui-like virus 2*, its missing segment was identified by DIAMOND BLASTp, hence no estimated completeness or *ConS* could be assigned. In SRR19790906, we identified two missing segments of two viruses which demonstrated high completeness and *ConS*.

**Fig. 5.**
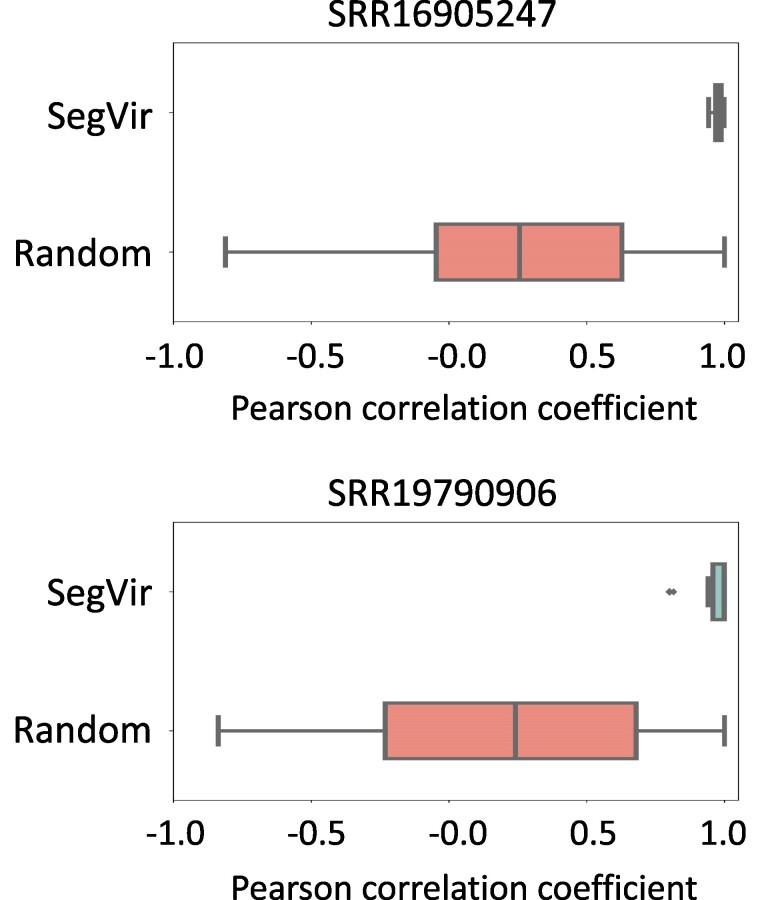
The correlation of contigs’ coverage distributions in the samples SRR16905247 and SRR2051466.

To provide further evidence that the identified segments indeed originate from the same virus, we calculated the correlation between identified viral contigs within the same virus. In order to obtain the coverage distribution of contigs, we downloaded additional SRA data of the same host from the corresponding sample project. Subsequently, we used the method in supplementary S1.1, Supplementary Material online to compute the correlation of the distribution. For SRR16905247 and SRR19790906, we downloaded nine and nine additional samples from PRJNA778885 and PRJNA851532, respectively, resulting in a coverage distribution length of 10 for each sample. All the SRA accession numbers used can be seen in supplementary Table S6, Supplementary Material online. We calculated the correlation among contigs within the same virus and randomly selected 100 contigs for comparison, as shown in Fig. 5. We can see that the correlation between contigs within the identified viral contigs was higher than that observed among randomly selected contigs, indicating that the identified segments highly possibly originate from the same virus. To better demonstrate the coverage distribution, we use the identified genomes of *Enontekio phenui-like virus 2* in SRR16905247 as an example to show the coverage distribution of its different segments, as shown in supplementary Fig. S6, Supplementary Material online.

### Performance in Identifying the Segmented RNA Viruses from a Set of Real Metatranscriptomes

We further evaluated the performance of SegVir using a collection of real metatranscriptomes obtained from mosquitoes. This dataset was generated by Mang Shi et al., encompassing samples from various locations and dates. We referred to samples gathered from the same date and location as a *group*. We conducted tests on three groups of samples, totaling 12 samples. Since each group of samples comprised multiple sequencing datasets, coassembly was performed on each group using MEGAHIT (Li et al. 2015). The details of each group are demonstrated in Table 4.

**Table 4:** The detailed information of assembled contigs in each group

Group ID	# Samples	N50	# Contigs	# Contigs (≥300 bp)
21NBJANKW	6	655	312,162	276,962
21NBJUNKW	4	710	128,441	125,389
21NBJUNYW	2	684	168,044	165,555

# Samples, the number of samples in the group; # Contigs, the number of coassembled contigs; # Contigs (≥300 bp), the number of contigs whose lengths are over 300 bp.

We applied the SegVir on the real metatranscriptomes, and the results for three groups of samples are shown in Table 5. Based on the identification results, we found that the viruses identified in the three samples originate from five families: *Birnaviridae*, *Orthomyxoviridae*, *Partitiviridae*, *Peribunyaviridae*, and *Phasmaviridae*. In some families, we detected two distinct viral RdRps. For instance, in the *Orthomyxoviridae* family of sample 21NBJUNKW, we identified both *Wuhan Mosquito Virus 4* (WhMV4) and *Wuhan Mosquito Virus 6* (WhMV6). For the identified viruses, all of them have an estimated completeness of 1 and are mosquito-borne. Furthermore, it is worth highlighting that WhMV6 is present in all the groups, which aligns with the findings of Ren et al. (2021) who also discovered its prevalence in mosquitoes sampled in Wuhan, suggesting its potential as a member of the core viromes in mosquitoes.

**Table 5:** The identified segmented RNA viruses in real data

Group	Family	Virus	*FunC*	*ConS*	*Ref ConS*
21NBJANKW (six samples)	*Birnaviridae*	*Aedes birnavirus*	1.0	2.0	2.0
	*Orthomyxoviridae*	*Wuhan Mosquito Virus 4*, *Wuhan Mosquito Virus 6*	1.0	4.02	4.02
	*Peribunyaviridae*	*Bunyaviridae environmental sample*	1.0	1.02	2.89
	*Phasmaviridae*	*Culex phasma-like virus*	1.0	2.07	2.07
21NBJUNKW (four samples)	*Birnaviridae*	*Mosquito X virus*, *Culex Y virus*	1.0	2.0	2.0
	*Orthomyxoviridae*	*Wuhan Mosquito Virus 4*, *Wuhan Mosquito Virus 6*	1.0	4.02	4.02
	*Phasmaviridae*	*Culex phasma-like virus*	1.0	2.0	2.0
21NBJUNYW (two samples)	*Orthomyxoviridae*	*Aedes orthomyxo-like virus 2*, *Wuhan Mosquito Virus 6*	1.0	4.02	4.02
	*Partitiviridae*	*Culex tritaeniorhynchus partitivirus*	1.0	2.0	2.0
	*Peribunyaviridae*	*Fangshan bunya-like virus*	1.0	1.02	2.89
	*Phasmaviridae*	*Barstukas virus*, *Aedes phasmavirus*	1.0	2.05	2.05

Family, the family of identified viruses; Virus, the names of viruses determined based on the RdRp segments; *FunC*, the completeness of the virus; *ConS*, the conservation score of the virus; *Ref ConS*, the reference conservation score.

**Fig. 6.**
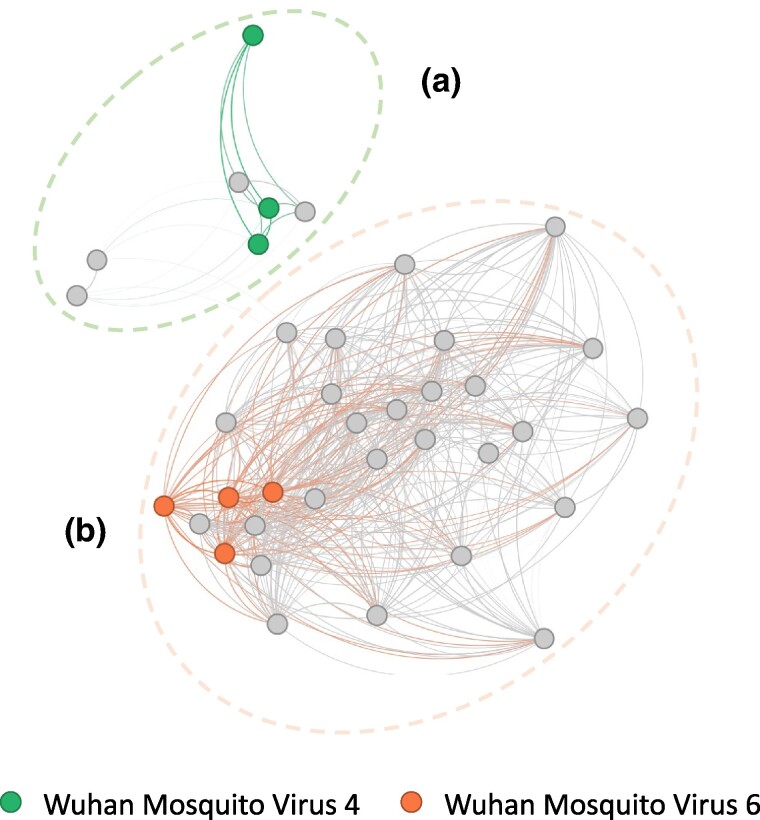
The similarity graph of *Orthomyxoviridae* contigs in 21NBJANKW. The colored nodes are used to represent the identified RdRp segments with lengths exceeding 1,000 bp, where different colors represent different viruses.

While most families contain only one virus, in the *Orthomyxoviridae* of sample 21NBJANKW, we identified two distinct RdRp segments. However, in this scenario, directly extending the taxonomy of the RdRp segments to other contigs for taxonomic determination becomes challenging. To address this, we first used the six samples within this group to obtain the coverage distributions for the contigs. Then, we treated the contigs as nodes, with the coverage distribution coefficient between contigs serving as edge weights for constructing a graph. Finally, we employed the OpenOrd (Martin et al. 2011) algorithm to arrange the distribution of nodes, where the higher the coefficient between points, the closer they are. The similarity graph is depicted in Fig. 6. We can see that the contigs clustered into two distinct groups: A and B, which contain RdRps from WhMV4 and WhMV6, respectively. Notably, the number of contigs in Group B far exceeds the maximal number of viral segments in *Orthomyxoviridae*. One possible reason for this phenomenon is the low coverage of segments, which causes segments to be assembled into multiple shorter contigs. Another potential reason is the presence of defective influenza viruses (Wang et al. 2020) in the sample, which lack their own RdRp and rely on the RdRp of other viruses for replication, or the occurrence of viral recombinants (Simon-Loriere and Holmes 2011), leading to a higher number of assembled non-RdRp contigs compared to RdRp contigs. Additionally, some RNA viruses produce subgenomic mRNAs during transcription (Sagan and Weber 2023), which may also result in segments being assembled into separate contigs.

We also discovered several segments missing from the NCBI database within the identified viruses. For instance, in sample 21NBJANKW, we identified a contig encoding the HA protein of WhMV4—a segment absent from the NCBI database. This contig exhibits a chimerical structure, where the protein encoded in the region [27, 1,442] is aligned to a HA HMMER model with an *e*-value of 1.5e−14, and the region [1,900, 3,530] aligns with host genomes with an *e*-value of 0.0 and identity of 98.5%. Furthermore, within the *Peribunyaviridae*, we identified a contig encoding a polyprotein, which is absent in the *Bunyaviridae* environmental sample within the database. Moreover, the difference between *ConS* and the *ref ConS* indicates that it is a relatively novel virus with regard to the reference database.

## Discussion

The identification and recovery of complete genomes of segmented RNA viruses from metagenomes are crucial for understanding their diversity, evolution, and impact on public health. In this study, we developed a tool named SegVir to address the challenges associated with identifying and retrieving segmented RNA viruses from complex metagenomes, and estimating the completeness. To evaluate the performance of SegVir, we conducted benchmark experiments using simulated and real metatranscriptomes. Our tool demonstrated high sensitivity and precision in identifying segmented RNA viruses, distinguishing them from nonsegmented RNA viruses, hosts, and bacteria infected by segmented RNA phages. We also compared SegVir with existing taxonomy classification tools, highlighting its ability to provide precise taxonomy information for queried contigs.

However, there are still some limitations that need to be addressed. In some cases, the data may contain multiple segmented RNA viruses within the same family, which do not exhibit high similarity with the reference database. Consequently, achieving precise contig binning at a high-resolution level, such as species level, becomes challenging. When the different segmented viruses possess different abundances and thus distinguishable coverage, a solution that takes advantage of the coverage distribution can be applied. Ideally, this method can benefit from multiple samples so that the trend of the coverage change of the segments from the same virus can be utilized for better genome reconstruction. However, using abundance as the key feature to bin viral contigs may not work well because their segments can accumulate to different abundances (Bonnamy et al. 2024). Moreover, when the number of samples is limited or the abundance of contigs is low, the calculated correlations between contigs may not be accurate. To better address this issue, in addition to calculating coverage distribution correlations, we can also incorporate features from nucleotide sequences. For example, the UTR-iBLASTn (Zhang et al. 2024) can determine whether segments belong to the same virus based on the similarity of their untranslated regions. We can first use SegVir to detect viruses in the samples, and then input the identified complete segmented RNA viral contigs into UTR-iBLASTn for contig binning.

Considering the feasibility and accuracy of data collection, SegVir’s current database only includes viruses from families known to contain segmented RNA viruses. For segmented RNA viruses without family annotation, manual retrieval, and validation are required, which is highly time-consuming, and thus those are not included in the current database. In the future, we will further expand our database by collecting viruses without family annotation. Additionally, we foresee that the increasing number of viruses will not significantly decrease the speed of SegVir. HMMER, the most computationally intensive part of SegVir, will mainly incur an increase in running time due to the diversity and number of HMMs rather than the underlying viruses. We believe that further expanding the database will enhance our capability to discover more novel viruses.

There are instances where the viral RdRp can be identified, but other segments cannot be detected. Due to the high diversity of some segments, sequence-homology-based methods may fail. For example, in experiments with simulated *Orthomyxoviridae* data, we found that the low similarity between the matrix and nonstructural proteins in the training and test data prevented SegVir from identifying segments containing these proteins. To address this issue, one potential solution is to use structural similarity. As noted in Daniel Marc’s review (Marc 2014), the structural similarity of the influenza virus’s nonstructural proteins is higher than their sequence similarity. However, predicting the structure of all proteins in a sample is currently impractical due to the significant time and computational resources required. A potential approach could be to quickly search for possible structures using databases like the ESM Metagenomic Atlas (Lin et al. 2023) and then compare the structural similarities to determine homology between sequences.

SegVir could also be extended to analyze large datasets to discover new segmented RNA viruses. For example, like the work by Edgar et al. (2022), who aligned extensive SRA data from NCBI to a RdRp database to identify novel RNA viruses, we need to maximize SegVir’s computational efficiency. To achieve this, we can perform preliminary analyses on samples, such as aligning reads to segmented RNA virus RdRp using Kaiju to determine the presence of segmented viruses and their taxonomy. Alternatively, we can use Serratus (Edgar et al. 2022) to directly obtain viral taxonomy information of the samples. If segmented viruses are confirmed, we can then assemble the samples into contigs and construct a database specific to the identified families for identifying the viruses. Additionally, we can consider using DIAMOND alone to search for viruses, sacrificing some sensitivity for increased efficiency. By streamlining the database and simplifying the search methods, we aim to efficiently identify new segmented RNA viruses in large datasets.

Finally, we provide some practical guidance for users. If users are attempting to identify novel viruses in samples, we recommend setting a more lenient alignment threshold (e.g. e-value=1e−5) when using SegVir. Because a lenient threshold may introduce a higher number of FPs, users may need to manually calibrate the results based on the information contained in the output files. SegVir also offers additional functions that allow users to set specific parameters (e.g. the length of identified contigs, the *e*-value for RdRp, etc.) to filter out FP from the results. In addition, users can modify the default parameters to suit specific circumstances. As described in the pipeline, the host sections in detected chimeric contigs will be removed by default. However, some studies have shown that viruses may naturally incorporate host genes during mutation (Nguyen et al. 2012; Paronetto et al. 2024). For such naturally occurring chimeric phenomena, the host sections should be retained. Therefore, the user can set the parameters in SegVir to choose whether to keep the complete chimeric contigs or not.

In future studies, it is essential to explore the correlations between different segments within the same virus. This analysis should encompass protein–protein correlations, sequence correlations (Newburn and White 2019), and phylogenetic tree correlations. By investigating these associations, a more comprehensive understanding of the relationships between viral segments can be achieved. Additionally, although SegVir has identified functional clusters of segmented RNA viruses, the functions of approximately half of these clusters remain unknown. Investigating these unknown functions will further our understanding of the biological properties of these viruses.

## Supplementary Material

msae171_Supplementary_Data

## Data Availability

The source code for the SegVir tool is available in the GitHub repository, which can be accessed at https://github.com/HubertTang/SegVir. This also includes the recovered segmented RNA viral genomes reconstructed from metatranscriptomes. The raw metatranscriptomic sequencing data utilized in this study are deposited in the NCBI Sequence Read Archive (SRA) under the accession numbers listed in supplementary Tables S6 and S7, Supplementary Material online.
