# Supplementary material for: SegVir: Reconstruction of Complete Segmented RNA Viral Genomes from Metatranscriptomes
Source: Mol Biol Evol. 2024 Aug 13;41(8):msae171. doi: 10.1093/molbev/msae171 (PMC11346362; doi:10.1093/molbev/msae171)
Supplement: msae171_Supplementary_Data [file msae171_supplementary_data.pdf]

SegVir: Reconstruction of complete segmented RNA viral genomes from metatranscriptomes

Supplementary materials

Contents

1 Supplementary Appendixes 1
- 1.1 Assign species-level taxon to contigs within the viral group 1
- 1.2 Compare the different methods of building the function cluster dataset 1
- 1.3 Identify the closest reference genomes in completeness estimation 4
- 1.4 Estimate the conservation score for viral genomes 4
- 1.5 Performance of leave-one-out test by family 5

2 Supplementary Figures 7

3 Supplementary Tables 9

1 Supplementary Appendixes

1.1 Assign species-level taxon to contigs within the viral group

For each viral group, we determine the taxonomy of contigs based on RNA-dependent RNA polymerase (RdRp). In most cases, a viral group contains only one virus. Consequently, we extend the taxon assigned to RdRp to the other contigs within the group. However, in rare instances, a group may contain RdRps from multiple viruses. In such cases, Segvir will compute the sequencing coverage correlation between non-RdRp and RdRp contigs to assign taxon. Our hypothesis posits that contigs originating from the same virus will exhibit similar depth distributions across multiple samples. As a result, we can assign the taxon of RdRp to non-RdRp contigs by assessing the correlation of their coverage distributions. In detail, we first map reads from each sample to the contigs and calculate their average depth within each sample to obtain the coverage distribution for each contig. Subsequently, we employ the Pearson correlation coefficient to measure the correlation of coverage distributions between pairs of contigs, with the formula as follows:

$$\text{correlation} = \frac{\sum(x-\bar{x})(y-\bar{y})}{\sqrt{\sum(x-\bar{x})^2(y-\bar{y})^2}} \tag{1}$$

where x and y are the coverage distributions of a pair of contigs. $\bar{x}$ and $\bar{y}$ are the mean of x and y, respectively. SegVir will calculate the coverages using CoverM [1] and output the distribution correlation between each non-RdRp contig and all RdRp contigs. Users can determine the taxon of the contigs with the alignment results. It is worth noting that the accuracy of this method is correlated with the number of samples, where larger datasets yield more accurate results.

1.2 Compare the different methods of building the function cluster dataset

To construct the function clusters (FCs) using protein clusters (PCs), we first computed the similarity between PCs and then employed graph clustering to group them into FCs. To accurately calculate the similarity between PCs, we tested three methods: sequence-based similarity, HMM profile similarity, and protein three-dimensional structure similarity. Suppose we have a pair of

PCs, denoted as $PC_i = \{s_1, s_2, ..., s_n\}$ and $PC_j = \{s'_1, s'_2, ..., s'_m\}$, where s' and s are proteins. Our goal is to compute the similarity between PC_i and PC_j, denoted as $sim(PC_i, PC_j)$. The formulas corresponding to the different methods are shown in Table S1.

Table S1: **The formulas of estimating the similarity between PCs using different methods.**

Method	Tool	Formula	Remarks
Sequence	DIAMOND	$\sum \frac{cov(s,s') \times ident(s,s')}{\lvert PC_i\rvert \times \lvert PC_j\rvert}$	cov and $ident$ denote the coverage and identity of alignment regions on the query, respectively.
profile HMM	hhalign [2]	$prob(hmm_i, hmm_j)$	hmm_i and hmm_j refer to the pHMMs corresponding to PC_i and PC_j, respectively. $prob$ denotes the probability of homology between these two profiles.
Structural similarity	ESMfold [3]	$\sum \frac{\text{tm-score}(\hat{s},\hat{s'})}{\lvert PC_i\rvert \times \lvert PC_j\rvert}$	$\hat{s}$ and $\hat{s'}$ represent the three-dimensional structures predicted by ESMFold for s and s', respectively. The TM-score [4] is used to calculate the similarity between these two structures.
	TM-vec [5]	$\sum \frac{\cos(v,v')}{\lvert PC_i\rvert \times \lvert PC_j\rvert}$	cos refers to the cosine similarity between two vectors.

We initially employ a sequence-based similarity method. We align the proteins in PC_i to proteins in PC_j using DIAMOND and define sequence similarity as the product of alignment coverage and identity. In addition, if there are multiple distinct alignments between a pair of sequences, we retain only the maximum score as their similarity. We compute the similarities of pairs of proteins and calculate the average similarity across all pairs as the measure of similarity between the PCs.

In addition to utilizing sequence similarity, we also evaluate the similarity between a pair of PCs by estimating the likelihood of homology between their corresponding Hidden Markov Model (HMM) profiles. We employ hhalign with default parameters to align the two HMM profiles, resulting in an estimated probability of homology between them.

Finally, we measure the similarity between PCs by assessing the similarity between the three-dimensional structures. Here, we use the Template Modeling Score (TM-score) as the metric, which primarily measures the overall similarity between two folds. The TM-score ranges from 0 to 1, and when the score is greater than 0.5, we consider the two proteins to have similar structures. We employ two methods to calculate the TM-score between two proteins. The first method involves using ESMFold to predict protein structures, followed by the use of tmalign to calculate the TM-score between the predicted structures. ESMFold is a high-precision protein structure prediction tool based on Evolutionary Scale Modeling (ESM). ESM is a protein foundation model trained on a large-scale protein data set using deep learning. The model demonstrates the ability to learn the evolutionary rules of proteins and the relationship between sequence-structure-function. For a single protein sequence, ESMFold can directly predict its atomic-level structure end-to-end. Moreover, tmalign is an algorithm used to compare protein structure similarity. It first identifies the optimal equivalent residues between two proteins based on structural similarity, and then outputs a TM-score value. Here, we use the average TM-score as the similarity between two PCs.

In the above method, predicting protein structures serves as an intermediate step is very time-consuming. Therefore, we also used a more efficient approach, using TM-Vec to directly calculate the TM-score between two protein sequences. TM-Vec is a deep learning model that integrates protein sequences and 3D structures during training, enabling the direct generation of vector embeddings containing structural information. For two proteins, s and s', we use TM-Vec to calculate their corresponding vector embeddings, v and v'. According to the literature, the TM-score between s and s' is equivalent to the cosine similarity between v and v'. After calculating the TM-score between the proteins of two PC groups, we compute the mean TM-score of all protein pairs as the similarity between the two PC groups.

We use the similarity calculated by the above methods to build the graph and cluster PCs,

Figure S1: **The evaluation of building edges and constructing clusters using different PC similarity estimation methods.** (a) The PR-curve of evaluating the accuracy of edge construction between different PCs. The value within the parentheses represents the area under the curve (AUC). (b) The bar plot of accessing the clustering using different types of edges. Different colors represent different tools for edge building.

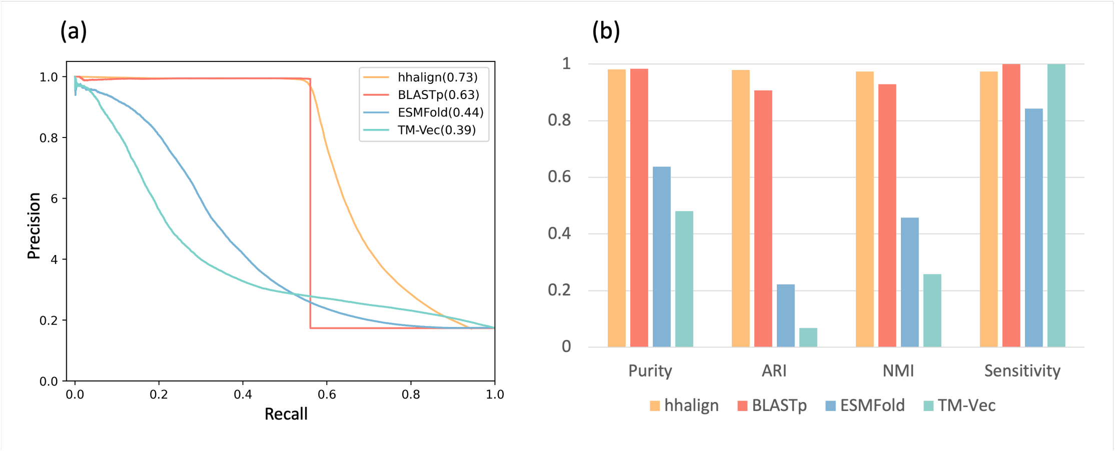

where node is the PC and edge is the similarity between PCs. To compare the performance, we first evaluated the accuracy of edge construction for different methods, followed by assessing the clustering results. We used 746 PCs derived from all proteins of the Orthomyxoviridae family as evaluation data. Each PCs was annotated based on the protein's description, with protein types including PA, PB1, PB2, neuraminidase, nucleocapsid, and so forth. We retained only the annotated PCs as the ground truth, totaling 739. In the graph, there should be edges connected between PCs that exhibit the same function. We employed the Precision-Recall (PR) curve to assess the performance, as shown in the Fig. S1 (a). Based on the area under the curve, the ranking of edge prediction accuracy is as follows: hhalign, BLASTp, ESMFold, and TM-Vec. At a recall of about 0.55, both hhalign and BLASTp exhibit very high precision, close to 1. However, as the recall increases, the precision of BLASTp drops sharply. Moreover, the accuracy of ESMFold is higher than that of TM-Vec.

After obtaining the similarity between PCs, we employ the Markov Clustering Algorithm (MCL) to cluster the PCs. We utilize purity, Adjusted Rand Index (ARI), Normalized Mutual Information (NMI), and sensitivity to evaluate the clustering results. The formulas are as follows:

$$\text{Purity} = \frac{1}{N}\sum_{i=1}^{K}\max_{j}|C_i \cap T_j| \tag{2}$$

$$\text{ARI} = \frac{\sum_{ij}\binom{n_{ij}}{2} - \left[\sum_i \binom{a_i}{2}\sum_j \binom{b_j}{2}\right]/\binom{N}{2}}{\frac{1}{2}\left[\sum_i \binom{a_i}{2} + \sum_j \binom{b_j}{2}\right] - \left[\sum_i \binom{a_i}{2}\sum_j \binom{b_j}{2}\right]/\binom{N}{2}} \tag{3}$$

$$\text{NMI} = \frac{2 \cdot I(C,T)}{H(C)+H(T)} \tag{4}$$

$$\text{Sensitivity} = \frac{TP}{TP+FN} \tag{5}$$

where the number of samples is N and the clustering result contains K clusters. $C = \{C_1, C_2, \ldots, C_K\}$ is the clustering result, and $T = \{T_1, T_2, \ldots\}$ is the actual clusters. n_{ij} denotes the number of samples in C_i and T_j, a_i and b_j denotes the number of samples in C_i and T_j. $I(C,T)$ is the mutual information, and $H(C)$ and $H(T)$ are the entropy of the C and T, respectively. TP is the number of true positive samples, FN is the number of false negative samples.

Among these metrics, purity is used to measure the accuracy of clustering, and both the Adjusted Rand Index (ARI) and Normalized Mutual Information (NMI) are utilized to evaluate the consistency between the predicted clusters and the actual clusters. Sensitivity is employed to access

the proportion of clustered samples. For all four metrics, a value closer to 1 indicates better performance. Finally, we utilized hhalign, BLASTp, ESMFold, and TM-Vec to obtain 17, 34, 28, and 7 FCs, respectively. The performance is shown in the Fig. S1 (b). Overall, hhalign demonstrated the best performance, followed by BLASTp, ESMFold, and TM-Vec. The purity of BLASTp was slightly higher than that of hhalign. However, the number of FCs generated by BLASTp was twice that of hhalign, resulting in lower ARI and NMI in BLASTp. While ESMFold's performance was still better than TM-Vec's, ESMFold had the lowest sensitivity among the four tools.

1.3 Identify the closest reference genomes in completeness estimation

When estimating the completeness of a query genome, we need to find the genome that is most similar to it in the reference genomes. Let s' be the FC set of the query genome and $S_{ref} = \{s_1, s_2, ..., s_n\}$ be the FC sets of all n reference viruses. To achieve this, we first need to calculate the distance between s' and each set s_i in S_{ref}. Here, we employ the weighted Jaccard similarity coefficient (J), as shown in the following function:

$$sim(s', s_i) = J(s', s_i) * \frac{s' \cap s_i}{|s'|} \tag{6}$$

$$J(s', s_i) = \frac{s' \cap s}{s' \cup s}, i \subseteq \{1, 2, ..., n\} \tag{7}$$

Here, we calculate the Jaccard similarity coefficient between s' and s_i, denoted as $J(s', s_i)$. We aim to identify the closest reference containing FCs that match those in the query while maximizing the J. To achieve this, we multiply $J(s', s_i)$ by the ratio of the intersection of s' and s_i to the size of s' (i.e., $|s'|$). This approach ensures that the most similar s_i includes as many elements from s' as possible, thereby avoiding overestimating completeness. For example, the FC set of the query genome G_q is {A, B, C}. In the reference database, there are two complete genomes, G_1 and G_2, corresponding to FC sets {A, B, D} and {A, B, C, D, E, F, G}, respectively. We can see that G_2 should be used to estimate the completeness of G_q, as the latter is a subset of the former. If we rely solely on the Jaccard coefficient, we would incorrectly identify G_1 as the closest genome to G_q, resulting in an overestimation of completeness. However, by employing Formula 6, we correctly identify G_2 as the closest genome to G_q and accurately estimate its completeness.

1.4 Estimate the conservation score for viral genomes

To calculate the conservation score, we first determine the weights of different function clusters (FCs). For RNA viruses, the RdRp plays a crucial role and is commonly used in evolutionary analyses. And viruses carrying distinct RdRps usually share low homology, implying differences in FC sets between viruses containing different RdRp FCs can be very large. Consequently, we group viruses based on the RdRp FCs, ensuring that viruses with the same RdRp FC are grouped together.

Assume the reference complete genomes are $G = \{g1, g2, g3, ...\}$, and we obtained the FC sets corresponding to each genome by aligning these genomes to the reference database. Within this group of viruses, we calculated the weight w for each FC by assessing its frequency across all genomes. The formula for calculating the weight is: $w_i = \#$ of genomes containing $FC_i / |G|$.

This implies that the more frequently FC_i occurs, the more conserved it is within this group of viruses, resulting in a weight w_i closer to 1. Consequently, for a query genome, after aligning it to obtain its corresponding FC sets, we first check which RdRp FCs are included. Subsequently, assuming that proteins in the genome can be aligned to n FCs, where w_i represents the weight of FC_i corresponding to specific RdRp, the conservation score is calculated by $\sum_{i=1}^{n} w_i$. The more conserved FCs a genome contains, the larger the cumulative score is, indicating the higher conservation between the genome and the reference database. When a query genome contains multiple RdRp FCs, we calculate corresponding scores for each RdRp FC and select the maximum score as the genome's conservation score ($ConS$). After obtaining the weights associated with the FCs, we compute the reference $ConS$ for each reference FC set. For a query genome, a smaller difference between the calculated score and the reference score indicates stronger conservation relative to the reference genomes.

1.5 Performance of leave-one-out test by family

To evaluate SegVir's ability to detect novel segmented viruses, we designed a leave-one-out test by family. We used complete genomes from 40 families containing segmented RNA viruses from the RefSeq database as our dataset. In each test, we selected one family as the test set and used the remaining 39 families to create SegVir's reference database. The experiment first evaluated the sensitivity of virus detection, where a virus was considered detected if at least one of its RdRp segments was identified. Next, we calculated the sensitivity for detecting non-RdRp segments among the identified viruses. Finally, we determined the proportion of detected viruses that could be identified by other families within the same order. This metric evaluates whether the virus detection is easier if the reference database contains families from the same order. The results are shown in Table S2.

As shown in Table S2, we can see that most viruses can be identified by other families within the same order, demonstrating SegVir's capability to detect new viruses from families not included in the sample. This is primarily due to the high conservation of RdRp, as evidenced by the significantly lower sensitivity for non-RdRp segments compared to RdRp. Although SegVir itself cannot determine whether the detected new virus belongs to a new family, users can construct a phylogenetic tree based on the identified RdRp to make further assessments.

Table S2: **The basic information of the dataset and the experimental results.**

	# genomes	Recall	Non-RdRp	Order
Alphatetraviridae	2	1	1	1
Alternaviridae	7	0.714	0	1
Amnoonviridae	1	1	0	1
Arenaviridae	61	0.967	0.015	1
Aspiviridae	3	1	0.428	nan
Benyviridae	5	1	0.5	0.8
Birnaviridae	13	0.462	0	nan
Bromoviridae	41	1	0.882	1
Chrysoviridae	30	0.933	0.144	0.964
Chuviridae	9	1	1	nan
Closteroviridae	14	1	1	1
Cruliviridae	3	1	1	1
Cystoviridae	7	0	0	nan
Discoviridae	3	1	0.5	1
Fimoviridae	26	1	0.179	1
Hadakaviridae	1	1	0.6	nan
Hantaviridae	37	1	0.972	1
Kitaviridae	11	1	1	1
Leishbuviridae	1	1	0.5	1
Mayoviridae	5	1	0.6	1
Megabirnaviridae	3	1	0	1
Nairoviridae	46	1	1	1
Nodaviridae	14	0.786	0	nan
Orthomyxoviridae	25	1	0.163	1
Partitiviridae	76	1	0.035	1
Peribunyaviridae	149	1	0.530	1
Phasmaviridae	31	1	0.714	1
Phenuiviridae	144	1	0.926	1
Picobirnaviridae	6	1	0.166	1
Polymycoviridae	10	1	0.775	nan
Potyviridae	6	1	0.833	nan
Qinviridae	8	0.875	0	nan
Quadriviridae	1	1	0.333	1
Rhabdoviridae	10	1	0	nan
Secoviridae	83	1	0.712	nan
Sedoreoviridae	42	0.190	0.012	1
Spinareoviridae	45	0	0	1
Tospoviridae	27	1	1	1
Virgaviridae	18	1	0.869	1
Yueviridae	2	1	0.5	nan

genome: the number of genomes included in each family. Recall: the detection sensitivity of viruses. Non-RdRp: the detection sensitivity of non-RdRp segments. Order: the proportion of detected viruses that could be identified by other families within the same order, where nan means there's only one family with this order.

2 Supplementary Figures

Figure S2: **The distribution of FC weights for all families and the selected 13 families.** The X-axis is the family, and the Y-axis is the distribution of FC weights.

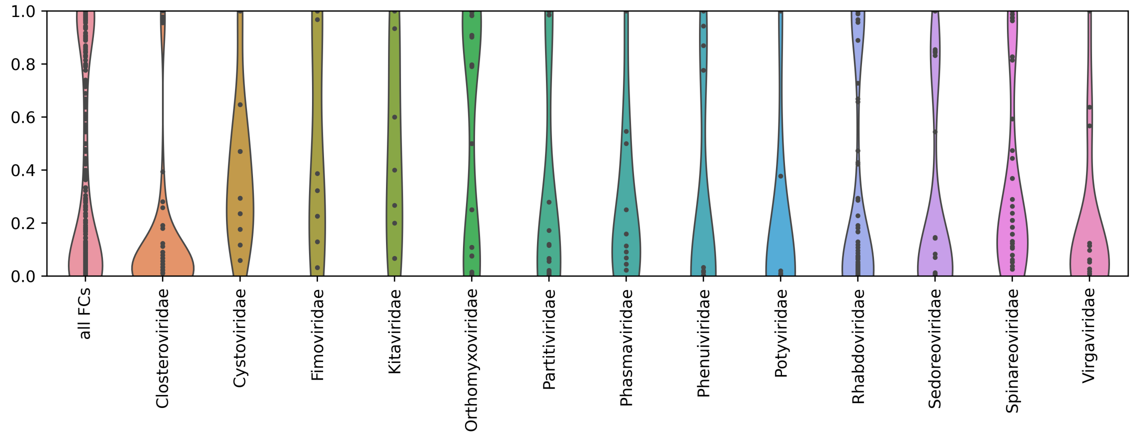

Figure S3: **The completeness estimation of identified genomes by different tools using the FC-based method.**

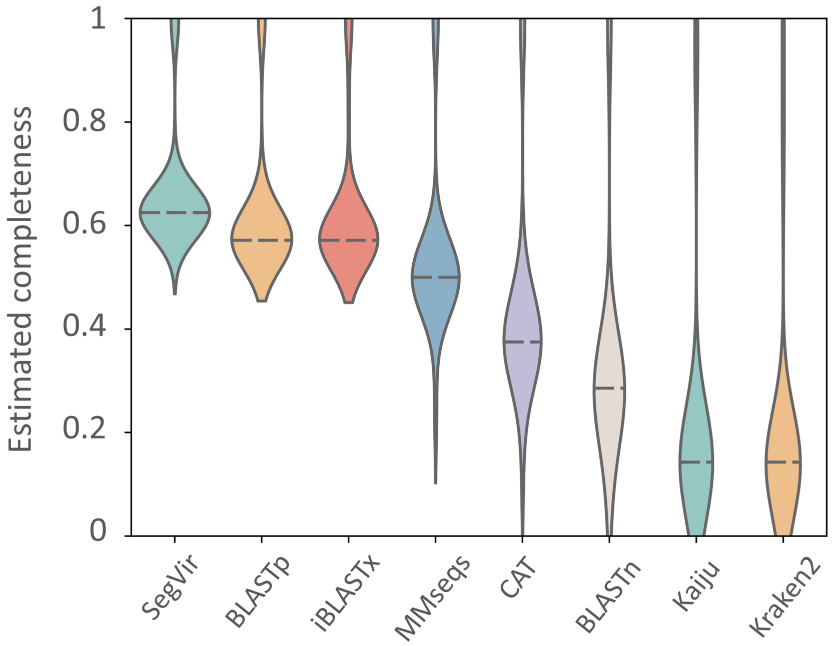

Figure S4: **The similarity between segmented RNA viruses and mosquito hosts.** We aligned segmented RNA viruses to different host genomes to analyze their similarities, where the X-axis represents the alignment length and the Y-axis represents alignment identity.

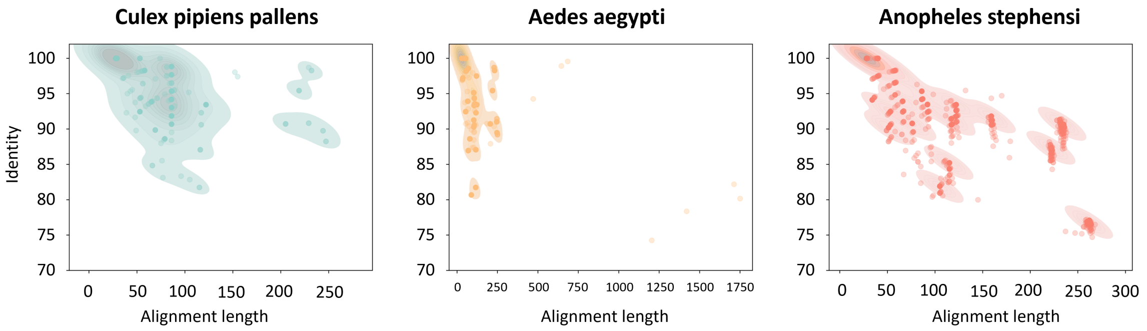

Figure S5: **The similarity between segmented RNA phages and bacteria.** We aligned segmented RNA viruses to different bacteria genomes to analyze their similarities, where the X-axis represents the alignment length and the Y-axis represents alignment identity.

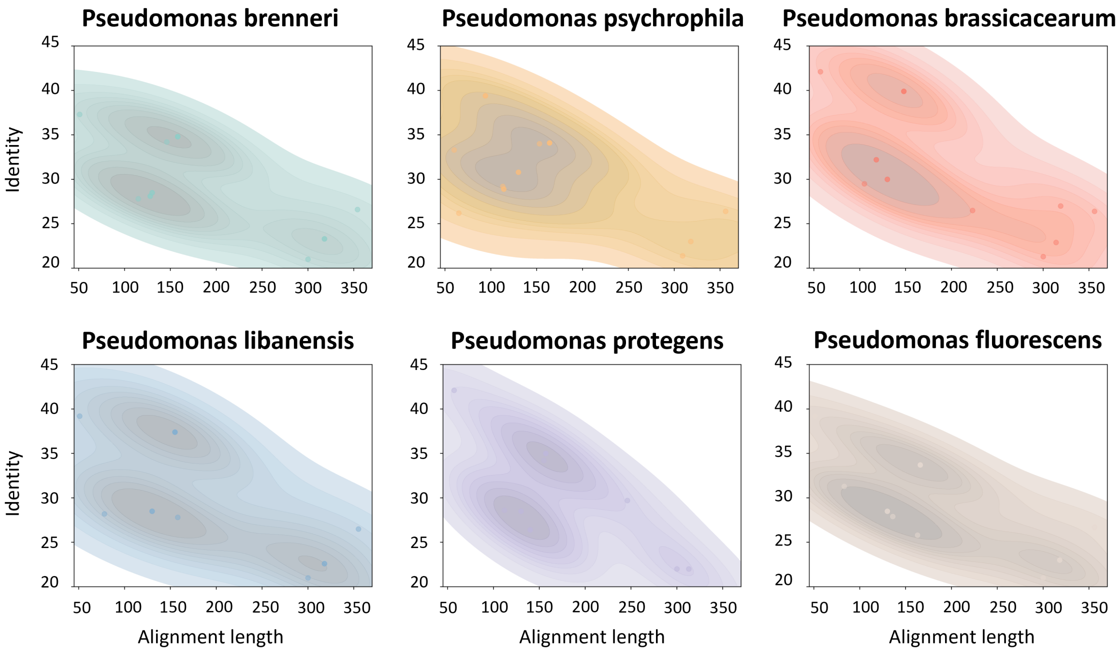

Figure S6: **The genomes and coverage distribution of *Enontekio phenui-like virus 2*.** (a) The identified genomes. The arrows indicate the proteins encoded on the segments, which encode RdRp and coat protein, respectively. (b) The coverage distribution of two segments across 10 samples.

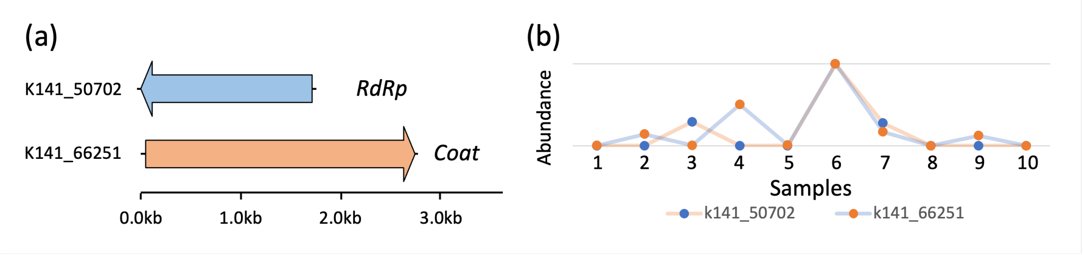

3 Supplementary Tables

Table S3: **The detailed information of the segmented RNA virus database.** The database is built using the viral proteins of 40 families containing segmented RNA viruses from the NCBI virus database. The reference FC sets and conservation score are constructed using the complete genomes from RefSeq.

family	# seg	# gene	# proteins	# PC	# FC
Alphatetraviridae	1-2	2-3	99	3	3
Alternaviridae	2-4	2-4	83	8	4
Amnoonviridae	10	10	535	7	5
Arenaviridae	2-3	2-4	7956	65	5
Aspiviridae	3-4	4-5	924	25	7
Benyviridae	2-5	7-10	2720	20	7
Birnaviridae	2	2-3	9245	56	4
Bromoviridae	3	4-6	11094	106	11
Chrysoviridae	3-7	3-7	603	36	8
Chuviridae	1-2	2-4	1311	40	5
Closteroviridae	1-3	5-14	25953	355	76
Cruliviridae	3	3	16	3	3
Cystoviridae	3	13-19	1654	55	27
Discoviridae	3	3	30	3	3
Fimoviridae	4-10	4-10	2889	61	11
Hadakaviridae	11	11	32	7	6
Hantaviridae	3	2-5	13891	158	4
Kitaviridae	2-4	5-9	1329	30	14
Leishbuviridae	3	3	82	6	5
Mayoviridae	2	3-5	465	14	6
Megabirnaviridae	2	4	48	5	3
Nairoviridae	2-3	2-3	5846	53	3
Nodaviridae	2	3-5	2719	82	8
Orthomyxoviridae	6-8	6-12	1500409	746	33
Partitiviridae	2-3	2-3	3479	143	24
Peribunyaviridae	3	3-5	7731	95	10
Phasmaviridae	3-4	2-5	1788	37	12
Phenuiviridae	2-8	2-13	17312	162	17
Picobirnaviridae	2	3-4	4952	227	8
Polymycoviridae	4-8	4-9	293	11	9
Potyviridae	1-2	2-3	34158	529	15
Qinviridae	2	2	64	8	2
Quadriviridae	4	4	33	9	4
Rhabdoviridae	1-2	2-14	63172	632	115
Secoviridae	1-2	2-4	5526	208	15
Sedoreoviridae	10-12	10-13	145195	664	56
Spinareoviridae	8-16	8-16	14575	226	64
Tospoviridae	2-3	3-5	8523	47	5
Virgaviridae	1-3	2-9	8878	159	26
Yueviridae	2	2	20	2	2

seg: number of segments. # gene: number of encoded proteins on each genome. # protein.: number of total proteins. # PC: number of protein clusters. # FC: number of function clusters.

Table S4: **The detailed information of the low-similarity dataset.**

Data	Viruses	# of genomes
Train	Influenza B virus	336
	Influenza A virus	2
	Isavirus salaris	1
	Quaranjavirus johnstonense	1
	Wellfleet Bay virus	1
	Lake Chad virus	1
Test	Influenza C virus	39
	Influenza A virus	4
	Influenza D virus	3
	Steelhead trout orthomyxovirus-1	1
	Rainbow trout orthomyxovirus-1	1
	Upolu virus	1
	Oz virus	1
	Tyulek virus	1

Table S5: **The details of the reference database of the simulated mosquito transcriptomic dataset.**

Type	Compositions
Host	Culex pipiens pallens, Aedes aegypti,Anopheles stephensi
Bacteria	Pseudomonas fluorescent, Pseudomonas protegens, Pseudomonas libanensis, Pseudomonas psychrophila, Pseudomonas brenneri, Pseudomonas brassicacearum
Segmented RNA virus	Culex Y virus, Port Bolivar virus, Culex mosquito virus 6, Mosinovirus, Nyando virus, Maprik virus, Wutai mosquito phasivirus, Phasivirus wutaiense
Non-segmented RNA virus	Yongsan picorna-like virus 4 ,Quang Binh virus ,Wenzhou sobemo-like virus 3 ,Hubei picorna-like virus 61 ,Hubei tombus-like virus 28 ,Culex Iflavi-like virus 1 ,Culex originated Tymoviridae-like virus ,Sanxia permutotetra-like virus 1 ,Jaagsiekte sheep retrovirus ,Wuhan Mosquito Virus 8 ,Negev virus ,Murine leukemia virus ,Hubei picorna-like virus 58 ,Zhejiang mosquito virus 1 ,Porcine type-C oncovirus ,Wenzhou tombus-like virus 11 ,Hubei virga-like virus 2 ,Culex Iflavi-like virus 4 ,Wuhan house centipede virus 9 ,Hubei picorna-like virus 66 ,Hubei arthropod virus 1 ,Hubei virga-like virus 21 ,Rhopalosiphum padi virus ,Hubei tetragnatha maxillosa virus 5 ,Culex mononega-like virus 2 ,Hubei tombus-like virus 27 ,Yongsan picorna-like virus 3 ,Getah virus ,Culex mosquito virus 4 ,Aphid lethal paralysis virus ,Zhejiang mosquito virus 3 ,Culex flavivirus ,Hubei permutotetra-like virus 1 ,Hubei virga-like virus 1 ,Japanese encephalitis virus ,Hubei mosquito virus 4 ,Yongsan iflavirus 1

Table S6: **The accession numbers of SRA data used to calculate the coverage distributions in experiments identifying the missing segments in the NCBI datasets.**

BioProject	SRA
PRJNA778885	SRR16905213<br>SRR16905214<br>SRR16905215<br>SRR16905216<br>SRR16905217<br>SRR16905218<br>SRR16905223<br>SRR16905231<br>SRR16905232<br>SRR16905247
PRJNA851532	SRR19790899<br>SRR19790900<br>SRR19790901<br>SRR19790902<br>SRR19790903<br>SRR19790904<br>SRR19790905<br>SRR19790906<br>SRR19790907<br>SRR19790908

Table S7: **The accession numbers of SRA data used to identify the segmented RNA viruses from real metatranscriptomes.**

Group	SRA
21NBJANKW	SRR27413683<br>SRR27413682<br>SRR27413681<br>SRR27413680<br>SRR27413689<br>SRR27413688
21NBJUNKW	SRR27413691<br>SRR27413690<br>SRR27413687<br>SRR27413685
21NBJUNYW	SRR27413686<br>SRR27413684

References

[1] Samuel T. N. Aroney, Rhys J. P. Newell, J. Nissen, Antonio P. Camargo, Gene W. Tyson, and Ben J. Woodcroft. CoverM: Read coverage calculator for metagenomics, 1 2024.

[2] Martin Steinegger, Markus Meier, Milot Mirdita, Harald Vöhringer, Stephan J Haunsberger, and Johannes Söding. Hh-suite3 for fast remote homology detection and deep protein annotation. *BMC Bioinformatics*, 20:1–15, 2019.

[3] Zeming Lin, Halil Akin, Roshan Rao, Brian Hie, Zhongkai Zhu, Wenting Lu, Nikita Smetanin, Robert Verkuil, Ori Kabeli, Yaniv Shmueli, et al. Evolutionary-scale prediction of atomic-level protein structure with a language model. *Science*, 379(6637):1123–1130, 2023.

[4] Yang Zhang and Jeffrey Skolnick. Scoring function for automated assessment of protein structure template quality. *Proteins: Structure, Function, and Bioinformatics*, 57(4):702–710, 2004.

[5] Tymor Hamamsy, James T Morton, Robert Blackwell, Daniel Berenberg, Nicholas Carriero, Vladimir Gligorijevic, Charlie EM Strauss, Julia Koehler Leman, Kyunghyun Cho, and Richard Bonneau. Protein remote homology detection and structural alignment using deep learning. *Nature Biotechnology*, pages 1–11, 2023.
